# Aged Mouse Cortical Microglia Display an Activation Profile Suggesting Immunotolerogenic Functions

**DOI:** 10.3390/ijms19030706

**Published:** 2018-03-01

**Authors:** Tanja Zöller, Abdelraheim Attaai, Phani Sankar Potru, Tamara Ruß, Björn Spittau

**Affiliations:** 1 Institute for Anatomy and Cell Biology, Department of Molecular Embryology, Faculty of Medicine, University of Freiburg, Freiburg 79104, Germany; tanja.zoeller@googlemail.com (T.Z.); abdelraheim.attaai@anat.uni-freiburg.de (A.A.); 2 Faculty of Biology, University of Freiburg, Freiburg 79104, Germany; 3 Department of Anatomy and Histology, Faculty of Veterinary Medicine, Assiut University, Assiut 71526, Egypt; 4 Institute of Anatomy, University of Rostock, Rostock 18057, Germany; PhaniSankar.Potru@med.uni-rostock.de (P.S.P.); TamaraHeike.Russ@med.uni-rostock.de (T.R.)

**Keywords:** microglia, aging, cerebral cortex, neuroinflammation

## Abstract

Microglia are the resident immune cells of the central nervous system (CNS) and participate in physiological and pathological processes. Their unique developmental nature suggests age-dependent structural and functional impairments that might contribute to neurodegenerative diseases. In the present study, we addressed the age-dependent changes in cortical microglia gene expression patterns and the expression of M1- and M2-like activation markers. Iba1 immunohistochemistry, isolation of cortical microglia followed by fluorescence-activated cell sorting and RNA isolation to analyze transcriptional changes in aged cortical microglia was performed. We provide evidence that aging is associated with decreased numbers of cortical microglia and the establishment of a distinct microglia activation profile including upregulation of *Ifi204*, *Lilrb4*, *Arhgap*, *Oas1a*, *Cd244* and *Ildr2*. Moreover, flow cytometry revealed that aged cortical microglia express increased levels of Cd206 and Cd36. The data presented in the current study indicate that aged mouse cortical microglia adopt a distinct activation profile, which suggests immunosuppressive and immuno-tolerogenic functions.

## 1. Introduction

Aging has been described as one of the major risk factors for development, onset and progression of several diseases, including cancer, cardiovascular pathologies, as well as neurodegenerative disorders [1]. In the central nervous system (CNS), age-dependent changes are believed to foster the development of neuropathologies including Alzheimer’s disease (AD) and Parkinson’s disease (PD). The high incidence of neurodegenerative diseases in elderly individuals has been linked to dysregulated functions of innate immune responses mediated by CNS-resident microglia [2]. Microglia develop from yolk sac progenitors in a PU.1- and interferon regulatory factor 8 (Irf8)-dependent manner [3] and further colonize the embryonic CNS parenchyma by chemotactic attraction driven by neuron-derived IL34 [4]. Sensing of neuronal IL-34 is mediated by colony stimulating factor-1 receptor (CSF1R), which is essential for microglia homing and migration towards the developing CNS [5]. After induction of a microglia-specific gene expression pattern [6] in a transforming growth factor β1 (TGFβ1)-dependent mechanism [7] and the establishment of the blood-brain barrier (BBB), microglia turnover in the adult CNS involves microglia proliferation and apoptosis [8], but not replacement by bone-marrow-derived progenitor cells [9,10]. This self-renewal capacity causing high proliferative activity of microglia has been hypothesized to result in telomere shortening and subsequent senescence of aged microglia [11]. However, it appears that microglia in aged mice do not develop telomere shortening, and only transgenic approaches, such as those described in the DNA repair-deficient *Ercc1* mutant mice, display accelerated aging associated with microglial senescence, dystrophy and functional impairments [12,13]. However, it has been reported that aged microglia exhibit enhanced expression of inflammatory markers tumor necrosis factor α (Tnfα), Il1β and Il6 after challenge with lipopolysaccharide (LPS), indicating an increased response of aged microglia [14], which is caused by age-dependent priming of microglia. Toll-like receptors Tl2, Tlr3, Tlr4 [15], as well as high-mobility group box 1 (Hmgb1) [16], have been described to be important mediators of microglia priming. Holtman et al. [17] recently identified a highly conserved gene expression profile of primed microglia, which significantly differed from gene expression patterns observed after LPS-driven acute microglia activation. Interestingly, RNA sequencing from aged total brain microglia revealed increased expression of genes involved in microglia-mediated neuroprotective effects [18].

In the present study, we addressed the age-dependent changes of cortical microglia in 24 months old C57BL/6 mice and observed decreased microglia numbers, enhanced expression of genes related to innate immune responses, and increased numbers of Cd206^+^ and Cd36^+^ microglia. Our data indicate that aging is associated with changes in microglia gene expression, which points towards activation of alternative markers and genes involved in immunosuppressive functions and immune toleration.

## 2. Results

### 2.1. Decreased Numbers of Cortical Iba1^+^ Microglia in Aged Mice

**Figure 1:**
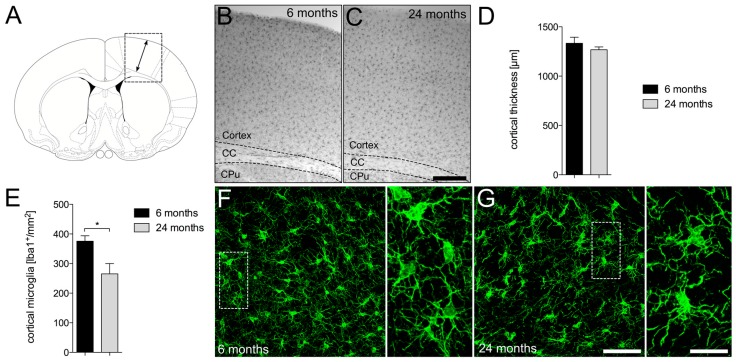
Age-dependent changes in cortical microglia. (**A**) Orientation scheme monitoring cortical areas for evaluation. Arrows mark the area for cortical thickness measurements. Representative images of Iba1^+^ microglia in cortices from 6-month-old (**B**) and 24-month-old mice (**C**). Scale bar represents 300 µm. Quantifications of cortical thickness (**D**) and cortical Iba1^+^ microglia numbers (**E**) are shown. Data are given as means ± SEM from three animals per age. *p*-value derived from Student’s *t*-test is * *p* < 0.05. Differences in morphology and distribution of Iba1^+^ microglia between 6-month-old (**F**) and 24-month-old (**G**) mice. Scale bars indicate 20 µm in overview images and 7 µm in high magnification detail images. CC = corpus callsosum, CPu = caudatoputamen.

In order to address age-dependent changes in cortical thickness and total microglia numbers in the frontal cortex of C57BL/6JRj mice, 50 µm vibratome sections were stained against the microglia marker ionized calcium binding adaptor molecular 1 (Iba1). Figure 1A displays the area used for analysis of cortical thickness. No obvious differences in cortical thickness were observed between young (Figure 1B) and aged (Figure 1C) mice. Moreover, quantification of thicknesses and statistical analyses also revealed no significant differences between young and aged mice (Figure 1D). Interestingly, quantification of cortical microglia (Iba1^+^) numbers revealed significant reduction of Iba1^+^ microglia in aged mice. Figure 1F displays microglia from young mice, showing normal ramifications with fine processes and homogenous distributions throughout the cortex (Figure 1F). However, aged cortical microglia presented a reduced branching pattern and an uneven distribution compared to microglia in young mice (Figure 1G). These data indicate that aging is associated with a reduction of cortical microglia numbers, which tend to cluster and thereby show an uneven distribution pattern.

### 2.2. Detection of a Distinct Gene Expression Profile in Aged Cortical Microglia

**Figure 2:**
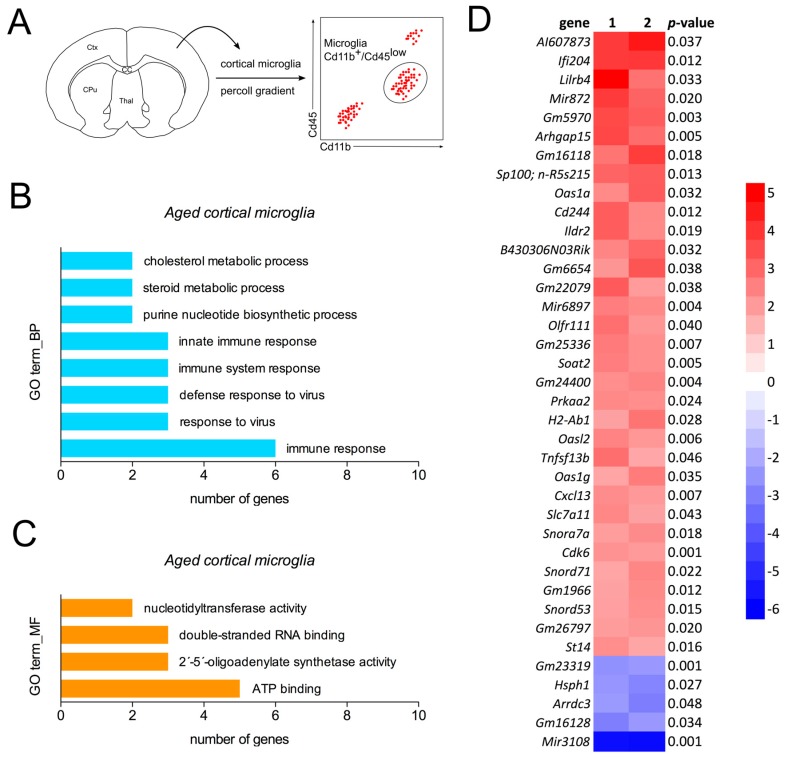
Gene expression pattern of aged cortical microglia. (**A**) Workflow scheme depicting microglia isolation and sorting strategy. (**B**,**C**) GO term enrichment analysis of biological processes (**B**) and molecular functions (**C**) as performed using DAVID Bioinformatics Resources 6.8. (**D**) Heatmap summarizing transcriptional changes in aged cortical microglia. Expression data from aged microglia are presented as log_2_-fold changes (*n* = 2) and compared to young (6-month-old) microglia. CC = corpus callosum, CPu = caudatoputamen, Ctx = cortex, Thal = thalamus.

As a next step, we addressed the transcriptional changes in aged cortical microglia. Therefore, frontal cortices from young and aged mice were dissected and microglia were isolated and stained for fluorescence activated cell sorting (FACS). Cd11b^+^/Cd45^low^-positive microglia were collected and RNA was isolated for cDNA microarrays (Figure 2A). Expression data were used for prediction of the biological processes (Figure 2B) and molecular functions (Figure 2C) of the top-regulated genes using the DAVID gene ontology (GO term) enrichment analysis. As shown in Figure 2B, immunological functions and cholesterol/steroid metabolic processes are predicted to be upregulated in aged cortical microglia. Activated molecular functions of aged microglia include ATP-binding, 2′-5′-oligoadenylate synthetase activity, double-stranded RNA binding and nucleotidyltransferase activity (Figure 2C). Data from cDNA microarray analysis revealed the upregulation of genes involved in immune responses including *Ifi204*, *Lilrb4*, *Arhgap15* and *Cd244*. Taken together, the data indicate activated immune responses and increased lipid metabolism in aged cortical microglia.

### 2.3. Increased Expression of Cd206 and Cd36 in Aged Cortical Microglia

**Figure 3:**
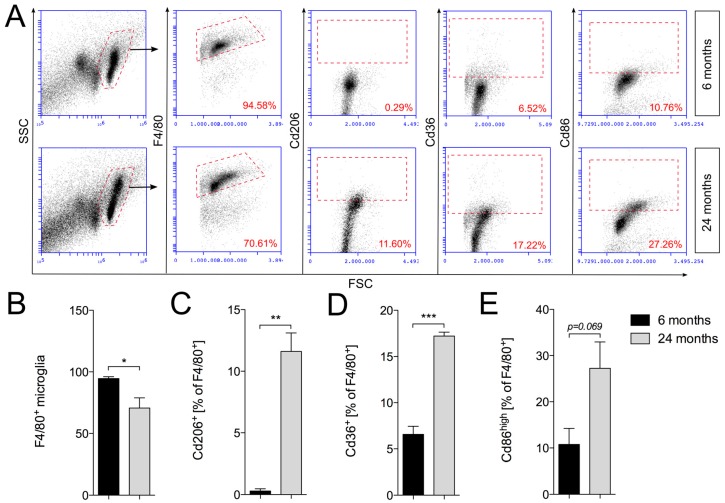
Expression of Cd206, Cd36 and Cd86 in F4/80^+^ cortical microglia. (**A**) Gating strategy and representative dot plots of Cd206^+^, Cd36^+^ and Cd86^high^ cortical microglia from young (6 months) and aged (24 months) mice. Quantification of (**B**) F4/80^+^, (**C**) Cd206^+^, (**D**) Cd36^+^ and (**E**) Cd86^high^ microglia. Data are given as percentages of F4/80^+^ microglia ± SEM from three animals per age. *p*-values derived from student’s *t*-test are * *p* < 0.05, ** *p* < 0.01 and *** *p* < 0.001.

Using flow cytometry, the expression of microglia activation markers Cd206, Cd36 and Cd86 was determined. Cortical microglia were isolated using the percoll gradient method and subsequently stained against F4/80 as a microglia marker in combination with either Cd206, Cd36 or Cd86. Flow cytometry revealed that total numbers of F4/80^+^ cortical microglia were significantly reduced in aged mice (Figure 3A,B), which confirms microglia quantifications depicted in Figure 1E. Moreover, we observed significant increases in Cd206^+^ (Figure 3A,C), as well as Cd36^+^ (Figure 3A,D) microglia. As given in Figure 3E, quantifications of F4/80^+^/Cd86^+^ microglia revealed that the percentages of Cd86^high^ microglia increase in the frontal cortices of aged mice; however, this increase did not reach statistical significance (*p* = 0.069). The results presented here demonstrate that aged cortical microglia display an activation pattern characterized by increased expression of M1-like as well as M2-like microglia activation markers. Based on the percentages of marker-positive microglia, it is highly likely that distinct cortical microglia subsets with specialized functions exist in aged mice. It further remains to be established to what extent these subsets contribute to anti-inflammatory and restorative functions in the aged cerebral cortex.

## 3. Discussion

In the present study, we demonstrate that aging is associated with distinct changes in cortical microglia. We describe that numbers of cortical microglia significantly decreased in 24-month-old male mice and that the remaining microglia in aged mice showed slight morphological changes, as well as an uneven distribution pattern. This observation is in congruence with a recent report showing that microglia numbers decreased in the nigrostriatal system and cortices of 18-month- and 24-month-old mice, respectively [19]. However, a different mouse strain was used in the present study, thus resulting in the necessity of ruling out differences in age-dependent changes of microglia numbers between mouse strains. Morphological changes of aged microglia, including retracted processes and reduced process branching, have also been reported in aged human neocortex samples [20], indicating functional changes described during aging [21]. However, morphological changes of microglia do not necessarily reflect their functional states and, thus, analysis of microglial gene expression has been widely used to gain insights into their functional changes during aging and in several disease models [17,18]. In order to understand the impact of aging on cortical microglia, we have isolated aged (24-month-old) Cd11b^+^/Cd45^low^ microglia and analyzed their gene expression profiles compared to young (6-month-old) microglia. Using cDNA microarray analysis, we demonstrated the upregulation of immune function related genes, including *Ifi204*, *Lilrb4*, *Arhgap15* and *Cd244*, and DAVID gene ontology (GO term) enrichment analysis further predicted the activation of innate immune responses in aged cortical microglia. However, detailed analysis of recent reports addressing the function of the above-mentioned genes suggests an immunoregulatory and/or anti-inflammatory phenotype of aged cortical microglia. However, the molecular functions of most of the observed upregulated genes are not understood in microglia and, thus, the prediction of potential microglia functions in the aged cerebral cortex can only be made based on recent reports addressing the role of these genes in other cell types. For instance, *Ifi204* expression has been described to increase in macrophages to inhibit proliferation and foster their differentiation [22]. Moreover, macrophage autophagy activation and IFN-β (interferon-β) release after bacterial infections has been linked to *Ifi204* expression [23]. Interestingly, the type I interferon IFN-β has been reported to limit CNS damage by abrogating chronic cytokine release. This functional feature of IFN-β might further explain its therapeutic potential in chronic autoimmune CNS pathologies such as multiple sclerosis [24]. The myeloid inhibitory receptor Lilrb4 (leukocyte immunoglobulin-like receptor b4), also referred to as Ilt3 (immunoglobulin-like transcript 3), Lir-5 (immunoglobulin-like receptor 5) or Cd85k, is a member of the leukocyte immunoglobulin-like receptor family (LILRs/LIRs), and represents an important mediator of immune tolerance [25]. Although the functions of LILRB4 in microglia are unknown and, thus, remain elusive, *Lilrb4*-deficiency has been demonstrated to result in increased Nf-κB (nuclear factor κB) signaling in atherosclerotic plaque-associated macrophages [26] and exaggerated LPS-induced cytokine/chemokine release from neutrophils [27]. In the CNS, increased expression of *Lilrb4* has been described in Cd11c^+^ microglia, which are believed to counteract amyloid deposition by increased amyloid β-uptake and degradation in a mouse model for AD [28]. Another interesting upregulated gene in aged cortical microglia is the Rho GTPase activating protein 15 (*Arhgap15*), which serves as a potent inhibitor of Rac1 (rac family small GTPase 1) [29]. The small GTPase Rac1 has been implicated in Nox2 (NADPH oxidase 2 or gp91^phox^)-mediated generation of reactive oxygen species (ROS) in microglia [30]. Notably, ROS generation via the Rac1-Nox2 axis has been demonstrated to be responsible for microglia-mediated neurotoxicity associated by Tnfα release and Nf-κB activation [31]. The SLAM (signaling lymphocytic activation molecule) receptor Cd244 was described to be essential in inhibiting the LPS-induced release of proinflammatory cytokines from splenic dendritic cells [32]. However, these functions in microglia have not been analyzed, so far. Taken together, the functional properties of the above-mentioned upregulated genes suggest a distinct phenotype of aged cortical microglia associated with regulation/inhibition of innate immune functions and anti-inflammatory and neuroprotective functions. This notion is further supported by increased surface expression of Cd206, also known as mannose receptor 1 (Mrc1) in aged cortical microglia. Cd206/Mrc1 expression was reported to be increased in alternatively activated microglia facilitating neuroprotective effects [33,34]. Furthermore, we provide evidence that the number of Cd36^+^ microglia was significantly increased in frontal cortices of aged mice. Recently, increased expression of the scavenger receptor Cd36 in microglia was linked to triggering receptor expressed on myeloid cells 2 (Trem2)-mediated alleviations of AD symptoms by enhanced uptake of Aβ and abrogation of memory loss [35]. Moreover, lack of Cd36 exacerbated injury in cerebral ischemia models associated with reduced engulfment of apoptotic neurons and enhanced Nf-κB signaling [36,37]. It remains elusive how microglia might promote neuroprotection in the aged brain, but expression and release of neuroprotective factors is likely to be one major mechanism. Release of insulin like growth factor 1 (Igf1) from microglia has recently been shown to be essential for survival of cortical layer V neurons during postnatal development [38] and could also be a candidate in the aged cerebral cortex. However, sophisticated proteome-based studies are necessary to understand which microglia-derived factors contribute to neuroprotective effects.

It has been described that microglia in different brain regions show distinct expression patterns of immunoregulatory markers, suggesting the existence of a huge immunological diversity between microglia from different functional CNS regions [39]. In the present study, we clearly demonstrate that cortical microglia in aged mice show a distinct activation profile, which is characterized by immunoregulatory and anti-inflammatory functions. These results suggest that aged microglia might support neuron survival rather than promoting detrimental effects on cortical neuron populations. However, upregulation of Cd86 further indicates M1-like microglia activation in aged mice suggesting a microglia activation phenotype fostering neurodegeneration. It remains to be established to what extent different functional microglia subsets are present in the aged murine cerebral cortex, and whether these cortical microglia phenotypes are region-specific or can be detected in other functional systems of the aged CNS. Finally, data from aged mice need to be compared with recently published reports addressing age-dependent changes in aged human microglia [40,41]. Together, the results presented here further broaden our understanding of age-dependent changes in cortical microglia functions and activation states, and indicate that aging alone—without any additional inflammatory trigger—does not necessarily result in a pro-inflammatory microglia activation.

## 4. Materials and Methods

### 4.1. Animals

All animal experiments in this study were approved by the Federal Ministry for Nature, Environment and Consumer’s Protection of the federal state of Baden-Württemberg (X-15/01A (9 February 2015), X-15/06A (1 December 2015), G-15/111 (3 December 2015)) and were conducted in accordance with the respective national, federal and institutional regulations. 6-month- and 24-month-old male C57BL/6JRj mice used for cortical microglia isolations (flow cytometry and RNA isolation) and immunohistochemistry were obtained from Janvier (Le Genest Saint Isle, France).

### 4.2. Microglia Isolation

Mice were deeply anesthetized by intraperitoneal injections of Ketamin (75 mg/kg)/Rompun (5.8 mg/kg) and transcardially perfused with ice-cold phosphate buffer solution (PBS). Brains were dissected and meninges were removed on absorbent paper. Brains were collected in cold buffer (1× HBSS (Hanks’ balanced salt solution) containing 1% BSA (bovine serum albumin) and 1 mM EDTA (ethylenediaminetetraacetic acid)), homogenized using a glass homogenizer and filtered through 75 μm cell strainers (Falcon). Samples were centrifuged 12 min at 300× *g* and 10 °C. For density gradient centrifugation, the pellet was resuspended in 5 mL 37% Percoll (P1644, Sigma Aldrich, St. Louis, MO, USA) in PBS, underlayed with 4 mL 70% Percoll and overlayed with 4 mL 30% Percoll in a 15 mL Falcon Tube (Corning, New York, NY, USA). Gradients were centrifuged for 40 min, 600× *g*, 10 °C, without breaks. Finally, the cell layer was collected from the 70% and 37% interface and transferred to PBS containing 1% FCS and centrifuged for 5 min, 200× *g*, 4 °C.

### 4.3. Flow Cytometry

Cells were stained with primary antibodies directed against Cd11b (1:20, 53-0112-82, eBiosciene, Thermo Fisher Scientific, Waltham, MA, USA), Cd206 (5 μL, FAB2535C, R&D Systems, Minneapolis, MN, USA), Cd36 (5 μL, MCA2748A647, AbD Serotech, BIO-Rad, Puchheim, Germany), Cd45 (1:20, 17-0451-82, eBiosciene), Cd86 (5 μL, MCA2463PE, AbD Serotech) and F4/80 (5 μL, MCA497A488, AbD Serotech) at 4 °C for 15 min. Fc (fragment crystallizable region) receptor blocking (TrueStain fcX, 101319, Biolegend, San Diego, CA, USA) was used to avoid unspecific antibody binding. Cells were washed and analyzed using the BD Accuri C6 flow cytometer (BD, Heidelberg, Germany).

### 4.4. Histology and Immunohistochemistry

Anesthetized mice were transcardially perfused using PBS followed by 4% paraformaldehyde (PFA). Brains were extracted and post-fixed in 4% PFA overnight. Free-floating 50 µm vibratome (Leica, Wetzlar, Germany) sections were incubated overnight with anti-IBA1 (1:1000, 019-19741, Wako, Japan). Alexa Flour 488-conjugated secondary antibodies (Cell Signaling Technology, Danvers, MA, USA) were used at 1:200 for 2 h at room temperature. Nuclei were counterstained using 4′6-diamidino-2-phenylindole (DAPI, Roche, Basel, Switzerland) and sections were mounted on glass cover slips. Imaging was performed using the Leica TCS SP8 confocal laser scanning microscope (Leica, Wetzlar, Germany) and LAS AF image analysis software (Leica, Wetzlar, Germany). For DAB (3,3′-diaminobenzidine) staining, peroxidase-conjugated secondary antibodies (Dianova, Hamburg, Germany) were used. Images were captured using A Zeiss AxioImager I (Zeiss, Göttingen, Germany) and the Stereoinvestigator Software 8.0 (MicroBrightField, Magdeburg, Germany).

### 4.5. Determination of Cortical Thickness and Cortical Microglia Numbers

Cortical thicknesses and microglia numbers were determined after immunohistochemical stainings (Iba1) of 50 µm coronal vibratome sections. After image acquisition using a Zeiss AxioImager I (Zeiss, Göttingen, Germany) and the Stereoinvestigator Software 8.0 (MicroBrightField, Magdeburg, Germany). Four serial sections were opened with ImageJ (National Institutes of Health, Bethesda, MD, USA), and the cortical thicknesses were analyzed after setting the scale for pixel/micron conversation using the ImageJ-integrated measurement function. Means were calculated from four serial sections, and cortical thicknesses are given in µm. Cortical microglia numbers were obtained using the automatic cell counting function of the ImageJ toolbox. Four serial sections were used and Iba1^+^ microglia were determined in a 0.67 mm^2^ rectangle. Microglia numbers were calculated for 1 mm^2^ and means from four sections per animals were plotted.

### 4.6. cDNA Microarray

Total RNA was extracted from Cd11b^+^/Cd45^low^ microglia after sorting with BD Cell Sorter FACS Aria Fusion or BD Cell Sorter FACS Aria III using the Picopure RNA extraction kit (Life technologies, Carlsbad, CA, USA) according to the manufacturer’s instructions. Cortices of three mice were pooled to obtain sufficient numbers of Cd11b^+^/Cd45^low^ microglia for RNA isolation. RNA quality was controlled using RNA pico chips on a Bioanalyzer 2100 (Agilent, Santa Clara, CA, USA). 2 ng total RNA was labeled using the GeneChip WT Pico Reagent kit (catalog number 902623, Thermo Fisher Scientific, Waltham, MA, USA) and hybridized to Affymetrix Mouse Transcriptome Array, MTA 1.0 (Affymetrix, Inc., Santa Clara, CA, USA). In this procedure, first strand cDNA was synthesized with a combination of a Poly-dT and random primers containing a 5′-adaptor sequence. A 3’-adaptor was added to the single-stranded cDNA followed by low-cycle PCR amplification. Next, the cDNA was used as a template for in vitro transcription (IVT), which produces amplified amounts of antisense mRNA (cRNA). The cRNA was then used as input for a second round of first-strand cDNA synthesis, producing single-stranded sense cDNA. Finally, the cDNA was fragmented using uracil-DNA glycosylase (UDG) and end-labeled with biotin and terminal deoxynucleotidyl transferase (TdT). The labeled targets were hybridized to GeneChip MTA 1.0 cartridge arrays, which were stained on a GeneChip Fluidics Station 450 and scanned on a GeneChip scanner 3000 7G (Thermo Fisher Scientific, Waltham, MA, USA).

### 4.7. Statistics

Data are given as means ± standard error of the mean (SEM). Two-group analysis (young vs. aged) was performed using Student’s *t*-tests. All statistical analyses were performed using GraphPad Prism 6 (GraphPad Software Inc., La Jolla, CA, USA) and *p*-values < 0.05 were considered as being statistically significant. To determine differential expression of genes in young and aged cortical microglia, the two-sample Bayesian *t*-test [42] was used.
